# The long-term neurodevelopmental outcomes of toddlers with SARS-CoV-2 infection in the neonatal period: a prospective observational study

**DOI:** 10.1186/s13052-024-01609-w

**Published:** 2024-02-27

**Authors:** Ezgi Yangin Ergon, Senem Alkan Ozdemir, Sinem Akbay Ak, Meltem Yenilmez, Buse Soysal, Oğuz Han Kalkanlı, Şebnem Çalkavur, Tülin Gokmen Yıldırım

**Affiliations:** 1 Clinic of Neonatology, T.C. Ministry of Health, Izmir Provincial Health Directorate, H.S.U. Dr. Behcet Uz Children’s Education and Research Hospital, 35210 Izmir, Turkey; 2 Clinic of Pediatrics, Developmental and Behavioral Pediatrics Unit, T.C. Ministry of Health, Izmir Provincial Health Directorate, H.S.U. Dr. Behcet Uz Children’s Education and Research Hospital, 35210 Izmir, Turkey

**Keywords:** Neurodevelopment, Newborn, Bayley-II, SARS-CoV-2

## Abstract

**Background:**

The effect of Severe Acute Respiratory Syndrome Coronavirus-2 (SARS-CoV-2) virus in the neonatal period on developing brain is still unknown. This study aims to investigate the long-term neurodevelopmental outcomes of newborns exposed to SARS-CoV-2 & Delta variant.

**Methods:**

At a tertiary referral center, a prospective observational cohort research was carried out. All babies who were equal to or more than 34 gestational weeks gestation and were admitted to the NICU between January 2021 and January 2022 due to SARS-CoV-2 infection (Delta - or Delta +) were included in the study. Infants who were hospitalized for non-SARS-CoV-2 reasons at similar dates and who had no history of invasive mechanical ventilation were incorporated as a control group using a 2:1 gender and gestational age match. Thirty infants were assigned to the study group and sixty newborns to the control group based on the sample size calculation. These toddlers’ neurodevelopment was evaluated between the ages of 18 and 24 months using the Bayley-II scale.

**Results:**

We enrolled 90 infants. SARS-CoV-2-positive infants had poorer psychomotor development index (PDI) scores and significantly greater mildly delayed performances (MDPs) at 18–24 months (PDI *p* = 0.05, MDPs *p* = 0.03, respectively). Delta variant showed statistically significant lower MDI and PDI scores (MDI *p*=0.03, PDI *p*=0.03, respectively). A smaller head circumference of SARS-CoV-2-positive toddlers was detected in the first year (*p < 0.001*), which improved at the second age.

**Conclusion:**

SARS-CoV-2-positive neonates revealed lower PDI scores and greater MDPs at 18th-24th months. The effect is most noticeable in Delta variant. Longer-term examination of neurodevelopmental outcomes and reevaluation of these children between the ages of 5 and 12 are critical.

**Supplementary Information:**

The online version contains supplementary material available at 10.1186/s13052-024-01609-w.

## Background

Respiratory viruses are opportunistic pathogens that infect the upper respiratory tract in humans and cause serious illness in particularly vulnerable populations. Some viruses can invade the central nervous system (CNS) and activate the immune system in the brain. A review reported by Koyuncu et al. about CNS viral infections concluded that viruses can reach the CNS under the right conditions, depending on viral (mutations in certain virulence genes) and host factors (immune suppression, age, and comorbidities). Immune events can cause long-term damage, similar to that seen in some neurodegenerative diseases [1].

Coronavirus disease 2019 (COVID-19), caused by the novel betacoronavirus (SARS-CoV-2), has become a global pandemic threat in the 21st century. The spectrum of diseases varies from asymptomatic infection to severe respiratory failure, septic shock, multiorgan failure, and death. COVID-19 is defined as a severe immunological consequence of SARS-CoV-2 infection. Most symptomatic cases present with fever, cough, and shortness of breath [2]. The potential involvement of COVID-19 in the CNS has attracted considerable attention due to neurological findings presented throughout the illness. Approximately one-third of the patients showed neurological symptoms, especially in those with a severe course of infection. Signal changes in the spleen of the corpus callosum on neuroimaging have been described in pediatric cases of severe acute respiratory syndrome from COVID-19 infection with pediatric multisystem inflammatory syndrome (MIS-C**)** [3]. Alcamo AM. and his colleagues reported 22% neurological symptoms such as smell and taste disturbances, headaches, and strokes after COVID-19 in hospitalized children [4]. Intracranial hypertension and acute encephalitis have been documented among children presenting with MIS-C [5, 6].

There are several potential mechanisms for the involvement of SARS-CoV-2 infection in the CNS. Animal studies suggest that angiotensin-converting enzyme II (ACE-II), which acts as a viral receptor, may mediate coronavirus-related neuronal damage. There is also evidence to argue that the virus can infect the cerebrovascular endothelium and brain. The virus causes apoptosis and necrosis in the brain parenchyma, particularly in the medial temporal lobe [7]. Proinflammatory cytokines can potentially alter epigenetic processes in the developing brain. Furthermore, postmortem studies of the human brain have provided evidence that human coronavirus variants and SARS-CoV-2 can infect both neurons and glia and that increased cytokine serum levels associated with SARS-CoV-2 infection indicate increased cytokine production [7]. The detection of antineuronal and antiglial autoantibodies in the cerebrospinal fluid of patients with SARS-CoV-2 and neurological symptoms increases the possibility that autoimmune mechanisms are associated with neurological involvement in the pathogenesis of SARS-CoV-2 [8]. Although neonatal encephalitis due to SARS-CoV-2 infection was rarely defined in the neonatal period, cranial MRI findings were found to be compatible with viral encephalitis [9].

Researchers are still monitoring what kind of symptoms SARS-CoV-2 infection produces in follow-up [10]. Currently, there is a lack of studies examining the impact of neonatal SARS-CoV-2 infection on the long-term neurodevelopment outcomes of affected infants. There are few studies on newborn neurodevelopment, particularly long-term studies [11]. Most of the follow-up studies are on infants born to mothers with SARS-CoV-2 infections [12–15]. In a study evaluating the routine management of newborns born to mothers with SARS-CoV-2 infection and their follow-up up to the first month, it was found that the risk of infection did not increase if the positive mother provided care to the newborn. It has been shown that breastfeeding and skin-to-skin contact, while adhering to protective measures, help babies’ growth and development in the first month and even provide better results [16]. Some researchers describe that neonates born to mothers with SARS-CoV-2 infection or who had a SARS-CoV-2 infection in the early neonatal period have a favorable neurodevelopmental outcome [17], while others reported neurodevelopmental sequelae and communication delay in some offspring [18].

This study aimed to evaluate the long-term neurodevelopmental outcomes of newborns exposed to SARS-CoV-2 and the delta variant by examining their cognitive, fine motor, gross motor, communication and social-emotional development skills with the Bayley-II Scale when they reached 18–24 months postnatally.

## Methods

A single-center prospective cohort study was performed at the Health Science University (HSU) Dr Behcet Uz Child Disease and Surgery Training and Research Hospital, Izmir, Turkey. This center is the largest referral neonatal center in Izmir and has a tertiary-level neonatal intensive care unit (NICU) with 60 incubators and approximately 1500 admissions annually. Neonates hospitalized for SARS-CoV-2 infection (Delta - or Delta +) between January 2021 and 2022 participated in the study.

### Ethics and consent

The study was approved by the institutional review board of the Dr Behcet Uz Child Disease and Surgery Training and Research Hospital Clinical Research Ethics Committee and strictly followed the institution’s ethical guidelines (Approval number 2023/06 − 03). Informed consent was obtained from the participants’ legal guardians.

### Study design

**Fig. 1:**
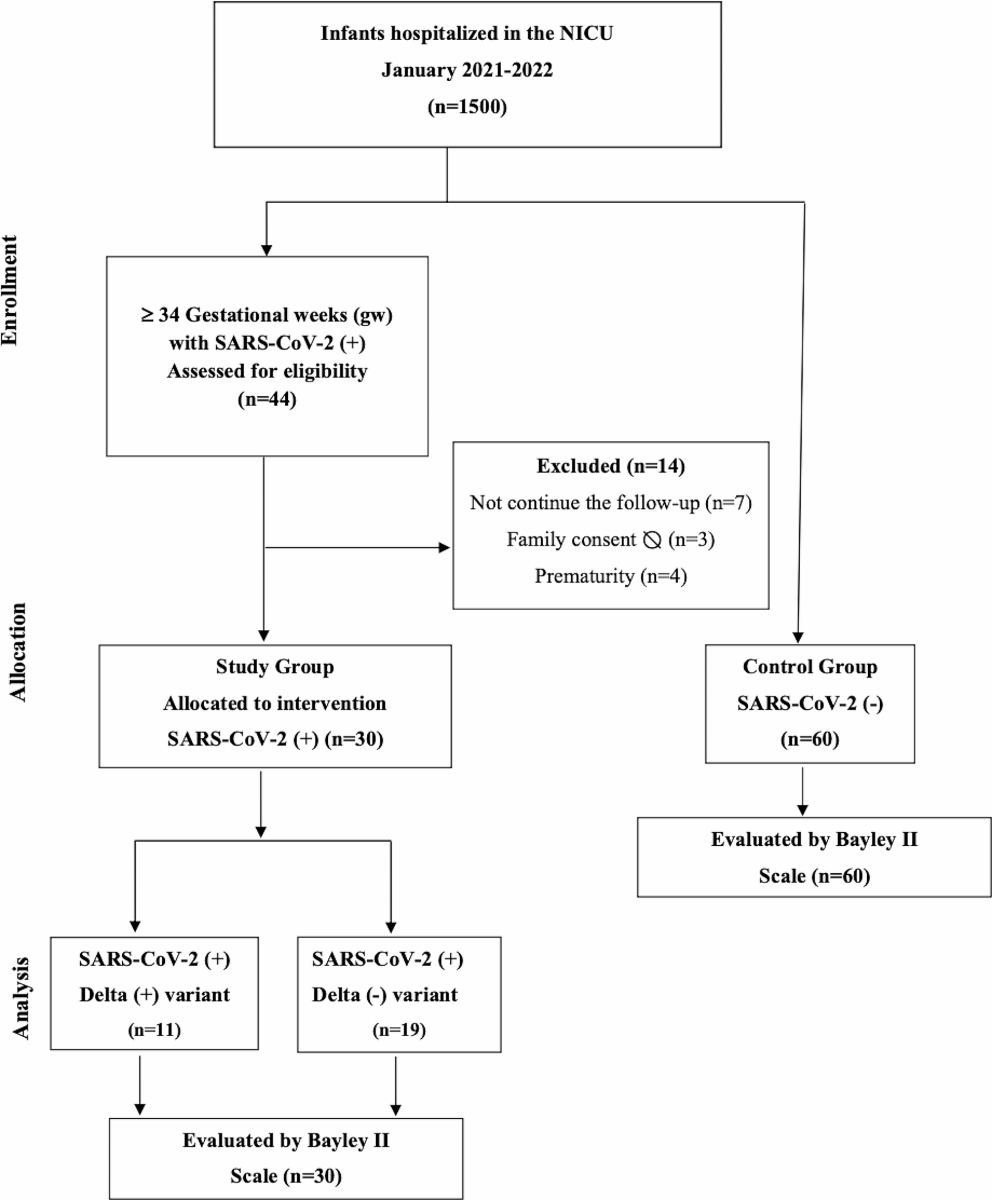
Flow chart for the selection of eligible infants in the study

Newborns equal to or greater than 34 gestational weeks hospitalized for SARS-CoV-2 infection (Delta - or Delta +) in the NICU between January 2021 and 2022 were included in the study. Newborns with premature birth (< 34 weeks of gestation), multiple pregnancies, major congenital anomalies, or hereditary neurometabolic disease were not included in the study. Moreover, infants whose family consent could not be obtained and who had insufficient data in the medical record system were excluded from the study. The flow chart of selected eligible infants in the study is presented in Fig. 1.

### Procedure

Oropharyngeal and nasal swab samples were obtained from infants who visited the outpatient clinic or emergency department with any SARS-CoV-2 symptoms or suspicion. Neonates exposed to SARS-CoV-2 infection in the early neonatal period who needed NICU admission were followed up in isolated rooms, and standard precautions were undertaken in accordance with the advice of the Infection Committee of the hospital. A real-time reverse transcription–polymerase chain reaction (RT‒PCR) test was performed by an experienced healthcare worker according to WHO recommendations. The delta variant was evaluated as a subgroup analysis in SARS-CoV-2-positive infants. The control group for non-SARS-CoV-2 reasons was selected by matching 2:1 according to gender and gestational age among babies who were hospitalized in a similar period, followed up with the same neonatal intensive care modalities, and had no history of invasive mechanical ventilation.

Complete blood counts, biochemical measurements (urea, creatinine, sodium, potassium, chlorine, AST, ALT, and total bilirubin), acute phase reactants, and chest radiographs of the infants were observed. Lumbar puncture was performed in infants whose fever (≥ 38 °C) lasted longer than 72 h and whose lung findings were not prominent. In the presence of a suspicious history or physical examination findings, further investigations were performed in terms of inflammation and organ involvement. An individualized and symptom-based approach to therapy was used. Cranial ultrasonography was used for neuroimaging.

Breastfeeding practices and discharge plans were carried out per the Turkish Neonatology Society’s (TNS) recommendations. Due to the potential risk of SARS-CoV-2 infection spreading from the mother’s respiratory secretions and breast milk, all parents were instructed to give their infants breast milk throughout the research period. However, it was later revised by the American Academy of Pediatrics to include breastfeeding [19].

### Data collection

Demographical and clinical characteristics of the infants, including maternal age and education, gestational week, gender, birth weight, length, and head circumference, were obtained from the electronic medical record system and the patients’ sheets. Clinical variables included 1st and 5th minute Apgar (appearance, pulse, grimace, activity, and respiration) scores, breastmilk intake during hospitalization, the need for oxygen support and mechanical ventilation, hearing test results, the need for physical therapy and/or special education, length of NICU stay, and rehospitalization status.

We monitored these babies closely for complications as possible MIS-C that may develop after discharge in the high-risk outpatient clinic weekly for 4 weeks. Using the phone numbers listed in the hospital system, the families of the toddlers were called when they were 18 to 24 months old, and an appointment for a neurodevelopmental evaluation was established.

### Neurological developmental assessment

Nancy Bayley created the Bayley Infant and Child Development Assessment Scale. The second edition of the Bayley Infant and Child Development Assessment Scale (Bayley-II) was released in 1993. In broad terms and in line with the child’s age, Bayley-II assesses the child’s developmental state. In the United States, children between the ages of 1 and 42 months are evaluated using the Bayley-II to track their development. It has been utilized in research and clinical practice for more than 40 years and has been standardized for 1700 children [20]. The Bayley-II scale is one of the most accurate measures of a child’s development. In a systematic review of the impact of early interventions on motor development, the Bayley scale was once again emphasized as the most popular way to evaluate neuromotor and developmental outcomes [21].

The mental development index (MDI) and psychomotor development index (PDI) are the evaluation scales for Bayley and Bayley-II. The mental development index examines linguistic and cognitive development, whereas the PDI evaluates gross and fine motor development [22].

The Bayley-II was used to assess long-term neurological and developmental outcomes between the 18th and 24th months. The toddlers’ Bayley and neurological assessments were carried out by the same two developmental and behavioral pediatric specialists. The presence of neurodevelopmental retardation was defined as cerebral palsy, bilateral hearing loss or blindness, or an MDI or PDI score less than 70, while mildly delayed performance (MDP) was defined as a score between 70 and 84 [23]. To the best of our knowledge, our outpatient clinics are overcrowded, appointment times are short, and the Bayley-III scale necessitates more testing. Therefore, Bayley-II was chosen for neurodevelopmental evaluation in this study.

### Sample size

The sample size consisted of patients who were hospitalized due to a SARS-CoV-2 infection in the NICU between January 2021 and 2022. By scanning the data from one year ago, the number of patients who were hospitalized in our hospital with similar characteristics and in a similar time period were evaluated, according to these evaluations, 30 patients were assigned to be included in the study.

### Statistical analysis

The SPSS 25.0 (IBM Corporation, Armonk, New York, USA) program was used in the analysis of variables. Parametric methods were used in the analysis of normally distributed numerical data, and nonparametric methods were used in the analysis of nonnormally distributed numerical data and categorical data. Kolmogorov‒Smirnov analysis was used to assess the normalization (*p* > 0.05 (sig.)). Descriptive statistics are presented as percentages and the mean ± standard deviation or median (interquartile range 25–75 percentiles) based on the normality of the data distribution. Chi-square test and Fisher’s exact test were used to compare the relationships of categorical variables with each other and Student’s t test was used to determine if the means of the data from two separate groups varied from one another (in the case of nonparametric data, the Mann‒Whitney U test was employed). A statistically significant difference between the categories was defined as *p* < 0.05.

## Results

### Demographic characteristics

Among the 1500 hospitalized neonates during the study period, forty-four (2.9%) were tested for SARS-CoV-2 (+). Fourteen babies were excluded from the study because of prematurity (*n* = 4), discontinued follow-up (*n* = 7), and a lack of consent (*n* = 3) to participate. A flow chart for the selection of eligible infants in the study is presented in Fig. 1. In eleven of these babies, the SARS-CoV-2 Delta variant (+) was identified. A total of 90 toddlers were evaluated, including 30 toddlers in the SARS-CoV-2 (+) study group and 60 toddlers in the SARS-CoV-2 (-) control group for neurodevelopmental outcomes. The antenatal, demographical, and clinical characteristics of the study population are summarized in Table 1.

**Table 1:** Demographic characteristics of the study groups

	Study Group SARS-CoV-2 (+) *(n = 30)*	Control Group SARS-CoV-2 (-) *(n = 60)*	*P*
GA (week) mean ± SDS	38.16 ± 0.98	38.16 ± 0.97	1.00
BW (g) mean ± SDS	3167 ± 390	3168 ± 302	0.98
Gender (Boy) n (%)	14 (46.6)	36 (60)	0.26
Delivery n (%) Vaginal Cesarean	8 (26.6)	38 (63.3)	***0.002***
	22 (73.4)	22 (36.7)	***0.002***
Prenatal history n (%) Preeclampsia GDM	1 (3.3)	4 (6.6)	0.66
	1 (3.3)	2 (3.3)	1.00
Maternal age (years) n (%) 15–25 25–35 >35	13 (43.3) 10 (33.3) 7 (23.3)	23 (38.3) 23 (38.3) 14 (23.3)	0.87
Maternal education n (%) Haven’t any education Elementery education High school University/postgraduate	5 (16.6) 11 (36.6) 9 (30) 5 (16.6)	9 (15) 23 (38.3) 17 (28.3) 11 (18.3)	0.99
APGAR score 5 (≥ 7) n (%)	30 (100)	60 (100)	NS
Fever ≥ 38 °C n (%)	14 (46.6)	16 (26.6)	0.44
Respiratory status n (%) Supplemental O_2_ Noninvasive respiratory support	4 (13.3) 3 (10)	3 (5) 6 (10)	0.37
Respiratory support (hour) mean ± SDS	34.85 ± 21.75	28.22 ± 13.20	0.46
Bayley II Scale age (month) mean ± SDS	20.50 ± 2.46	21.10 ± 2.46	0.30

GA: gestational age, BW: birth weight, GDM: gestational diabetes mellitus

In our research, there were no differences between the study and control groups in terms of antenatal and demographic findings, such as gestational week, birth weight, gender, prenatal history, maternal age, educational status, Apgar at 5 min, presence of fever at admission, and Bayley II test application time. SARS-CoV-2 (+) babies had significantly lower vaginal delivery percentages than SARS-CoV-2 (-) babies [SARS-CoV-2 (+) *n* = 8 (26%); SARS-CoV-2 (-) *n* = 38 (62%); *p* = 0.002] (Table 1). The cesarean section percentage in SARS-CoV-2 (+) babies is 73.4% (*n* = 22) and 36.7% (*n* = 22) in SARS-CoV-2 (-) babies, the cesarean section percentage is quite high in SARS-CoV-2 (+) babies. Twenty-two of the infants who tested positive for SARS-CoV-2 had symptoms such as fever, respiratory symptoms, and poor feeding. MIS-C was not detected in any of the patients.

### Neuroimaging & neurodevelopmental outcomes

When SARS-CoV-2-positive newborns were evaluated using cerebral ultrasonography at 1 month, 6 months, and 1 year of age, no abnormal findings were found. The mean Bayley-II Scale age (months) at evaluation was 20 months for SARS-CoV-2 (+) toddlers and 21 months for SARS-CoV-2 (-)toddlers and did not differ between study groups (*p* = 0.30). Low MDI or PDI scores were detected in 15 (50%) of the patients who tested positive for SARS-CoV-2, while they were seen in 6 (10%) of the patients who tested negative. Additionally, both low MDI and PDI scores were identified in 9 (30%) SARS-CoV-2-positive patients and in 5 (8.3%) SARS-CoV-2-negative patients. SARS-CoV-2 (+) newborns had significantly higher rates of mild delay performance at the 18th–24th months [SARS-CoV-2 (+) *n* = 13 (43%); SARS-CoV-2 (-) *n* = 6 (10%), *p* = 0.03]. According to the Bayley-II scale, SARS-CoV-2-positive newborns also showed delays in gross and fine motor development, and the PDI scores were lower than those of the controls [SARS-CoV-2 (+): 89.13 ± 7.49; SARS-CoV-2 (-): 92.30 ± 7.22, *p* = 0.05]. The verbal and cognitive development of the two groups did not differ statistically; nevertheless, the MDI scores were comparable (*p* = 0.64). Evaluations of children’s linguistic, cognitive, gross, and fine motor skills using the Bayley-II exam revealed that one child needed special education and another needed speech therapy. No incidences of blindness, deafness, or cerebral palsy were found in the long-term follow-up of toddlers with SARS-CoV-2 (+) infection. Two of the toddlers who tested positive for SARS-CoV-2 had positive neurodevelopmental retardation indices (NDIs, *p* = 0.12). Table 2 compares neurodevelopmental assessments between the study and control groups.

**Table 2:** Neurodevelopmental evaluation between study & control groups

	Study Group SARS-CoV-2 (+) *(n = 30)*	Control Group SARS-CoV-2 (-) *(n = 60)*	*P*
MDI mean ± SDS	88.51 ± 8.33	89.56 ± 12.82	0.64
PDI mean ± SDS	89.13 ± 7.49	92.30 ± 7.22	***0.05***
NDI* Positivity n (%)	2 (6.6)	0	0.12
MDP n (%)	13 (43.3)	6 (10)	***0.03***
Blindness n (%)	0	0	NS
Deafness n (%)	0	0	NS
CP n (%)	0	0	NS
Need for Physical Therapy n (%)	1 (3.3)	0	0.33
Need for Special Education n (%)	1 (3.3)	0	0.33

**MDI-** Mental developmental index, **PDI-** Psychomotor developmental index, NDI*- Neurodevelopmental retardation index defined as the presence of one or more of the following: (1) Moderate to severe cerebral palsy with functional losses, (2) Bilateral hearing loss and blindness, (3) Bayley-II MDI or PDI score < 70, **MDP-** Mildly delayed performance, defined as the presence of Bayley-II MDI or PDI score 70–84, **CP -** Cerebral palsy

In the delta subgroup analysis, low MDI or PDI scores were detected in 9 (81.8%) of the patients with the delta (+) variant and in 6 (31.5%) of the patients with the delta (-) variant. When Delta (+) and (-) variants were compared in terms of both low MDI and PDI scores, the number of Delta (+) patients was 5 (45.4%) and that of Delta (-) was 4 (21%). The subgroup analysis of SARS-CoV-2 (+) toddlers in terms of the delta variant showed significantly lower MDI and PDI scores in the delta (+) variants [MDI _delta (+)_: 86.36 ± 11.91; MDI _delta (−)_: 89.75 ± 12.82, *p* = 0.03; PDI _delta (+)_: 87.50 ± 5.11; PDI _delta (−)_: 90.05 ± 7.18, *p* = 0.03]. When we compared mildly delayed performance between Delta (+) and (-) SARS-CoV-2 toddlers, a significantly higher number of delayed MDP scores were detected in Delta (+) variants [MDP _Delta (+)_*n* = 9 (81%); MDP _Delta (−)_*n* = 4 (21%), *p* = 0.006] (Table 3).

**Table 3:** Neurodevelopmental evaluation according to SARS-CoV-2 delta variant

	SARS-CoV-2 (+) Delta (+) variant *(n = 11)*	SARS-CoV-2 (+) *Delta (-) variant (n = 19)*	*P*
MDI mean ± SDS	86.36 ± 11.91	89.75 ± 12.82	***0.03***
PDI mean ± SDS	87.50 ± 5.11	90.05 ± 7.18	***0.03***
NDI* Positivity n (%)	0	2 (10.5)	0.57
MDP n (%)	9 (81.8)	4 (21)	***0.006***
Blindness n (%)	0	0	NS
Deafness n (%)	0	0	NS
CP n (%)	0	0	NS
Need for Physical Therapy n (%)	1 (9)	0	0.63
Need for Special Education n (%)	1 (9)	0	0.63

**MDI-** Mental developmental index, **PDI-** Psychomotor developmental index, NDI*- Neurodevelopmental retardation index defined as the presence of one or more of the following: (1) Moderate to severe cerebral palsy with functional losses, (2) Bilateral hearing loss and blindness, (3) Bayley-II MDI or PDI score < 70, **MDP-** Mildly delayed performance, defined as the presence of Bayley-II MDI or PDI score 70–84, **CP -** Cerebral palsy

There was no statistically significant relationship between delta variant positivity and NDI scores, need for physical therapy, and/ or special education (*p* = 0.57, *p* = 0.63, *p* = 0.63, respectively)

### Secondary outcomes

The head circumference of the study group at 12 months was found to be smaller than that of the control group according to the secondary outcomes [HC^12 month^ SARS-CoV-2 (+): 45.90 ± 0.79 cm; SARS-CoV-2 (-): 46.63 ± 1.06 cm, *p* < 0.001], even though there was no significant difference between the groups in terms of head circumference from birth to 12 months (*p* > 0.05). However, by the time the child was 2 years old, this statistically significant difference had vanished (*p* = 0.70) (Table 4).

**Table 4:** Comparison of secondary outcomes between study & control groups

	Study Group SARS-CoV-2 (+) *(n = 30)*	Control Group SARS-CoV-2 (-) *(n = 60)*	*P*
Head circumference at birth (cm) mean ± SDS	34.98 ± 0.77	35.06 ± 0.82	0.64
Head circumference 1st month (cm) mean ± SDS	37.98 ± 0.77	38.00 ± 0.90	0.64
Head circumference 6th month (cm) mean ± SDS	43.76 ± 1.15	43.73 ± 1.14	0.89
Head circumference 12th month (cm) mean ± SDS	45.90 ± 0.79	46.63 ± 1.06	***< 0.001***
Head circumference 18th month (cm) mean ± SDS	47.76 ± 0.74	47.83 ± 1.01	0.72
Head circumference, 24th month (cm) mean ± SDS	49.56 ± 0.78	49.45 ± 0.82	0.70
Breastmilk intake time (/days) mean ± SDS	3.76 ± 1.38	5.31 ± 2.60	***0.05***
Hospitalization time (/days) mean ± SDS	10.73 ± 3.59	6.71 ± 3.58	***< 0.001***

The breastmilk intake time was shorter in SARS-CoV-2-positive babies than in controls [SARS-CoV-2 (+): 3.76 ± 1.38 day; SARS-CoV-2 (-): 5.31 ± 2.60 day, *p* = 0.05], and the hospitalization time was significantly longer [SARS-CoV-2 (+): 10.73 ± 3.59 day; SARS-CoV-2 (-): 6.71 ± 3.58 day, *p* < 0.001] (Table 4). As shown in Table 5, there was no statistically significant difference between Delta variant (+) and Delta variant (-) toddlers in terms of breastmilk intake time and head circumference length at 12 months (*p* = 0.15, *p* = 0.22, respectively). However, the hospitalization time was found to be longer in Delta (+) variant toddlers [Delta (+): 12.90 ± 1.64 day; Delta (-): 9.47 ± 3.83 day, *p* < 0.001].

**Table 5:** Comparison of secondary outcomes according to the SARS-CoV-2 delta variant

	SARS-CoV-2 (+) Delta (+) variant *(n = 11)*	SARS-CoV-2 (+) Delta (-) variant *(n = 19)*	*P*
Head circumference at birth (cm) mean ± SDS	35.09 ± 0.70	35.05 ± 0.91	0.30
Head circumference 1st month (cm) mean ± SDS	37.90 ± 0.94	38.05 ± 0.91	0.92
Head circumference 6th month (cm) mean ± SDS	43.63 ± 0.80	43.78 ± 1.31	0.14
Head circumference 12th month (cm) mean ± SDS	46.63 ± 0.80	46.63 ± 1.21	0.22
Head circumference 18th month (cm) mean ± SDS	47.63 ± 0.80	47.94 ± 1.12	0.41
Head circumference 24th month (cm) mean ± SDS	49.50 ± 1.00	49.42 ± 0.78	0.71
Breastmilk intake time (/days) mean ± SDS	4.09 ± 1.64	3.57 ± 1.21	0.15
Hospitalization time (/days) mean ± SDS	12.90 ± 1.64	9.47 ± 3.83	***< 0.001***

## Discussion

In our study, a control group of 60 term toddlers hospitalized for non-SARS-CoV-2 reasons and matched for sex and gestational age served as the comparison group for the neurodevelopmental outcomes of 30 toddlers with SARS-CoV-2 (+) in the neonatal period at 18–24 months of age. Compared to the control group, the long-term neurodevelopmental outcomes of SARS-CoV-2 (+) toddlers at 18–24 months who underwent Bayley-II assessments were affected.

The SARS-CoV-2 virus has neuroinvasive properties. Adult investigations have indicated that some adults with moderate or severe SARS-CoV-2 infection have a predominance of neurological symptoms, increased neuronal damage, and glial activation markers [24–26]. Evidence associating SARS-CoV-2 infection with brain abnormalities and cognitive problems in adulthood has since corroborated this theory [27–29]. Studies have shown that long-term COVID findings (persistent fatigue, headaches, dyspnea, concentration issues, depression, skin lesions, and gastrointestinal complaints that persist months after initial SARS-CoV-2 infection) are also detected in pediatric populations, similar to the adult population, although the pediatric population has a milder course of SARS-CoV-2 infection than adults [30]. In a survey study that involved 57 hospitals and 89 children with suspected COVID findings, fatigue was found in 45% of patients 2–12 months after acute infection, and severe functional limitations prevented children from attending school in 36% of patients [31]. Anosmia, lethargy, and headaches were long-term, persistent COVID neurological symptoms in a research assessing the long-term clinical state of 322 SARS-CoV-2 positive children between the ages of 1.5 and 17 years. Furthermore, it was shown that 12% of the patients in the 1.5–5 age group experienced internalization issues, while the 6–18 age group experienced long-term psychological and cognitive issues such anxiety and post-traumatic stress disorder [32]. In a systematic review of 176 neonatal SARS-CoV-2 cases, researchers reported that 18% of infants had neurological signs such as hypertonia, irritability, as well as hypotonia and lethargy, and apnea [33]. Additionally, in another report involving 5 neonatal SARS-CoV-2 cases, MRI showed various abnormalities in four; these were partially associated with reduced neurobehavioral scores [34]. In order to evaluate the general movement assessment in infants 3–5 months old, Aldrete Cortez et al. videorecorded them. They compared 28 unexposed controls to 28 babies delivered to moms who infected SARS-CoV-2 during the third trimester. Babies in the exposed group saw a 22% incidence of absentee or less fidgety movements, which may be a sign of brain impairment early on [35]. Our findings demonstrated that 50% of SARS-CoV-2-positive neonates had lower MDI or PDI scores. SARS-CoV-2-positive neonates additionally exhibited a 43% greater incidence of mild delay performance at the 18th-24th months than controls. Lower MDI or PDI scores were found in 81.8% of Delta-positive variants, while both lower MDI and PDI scores were found in 45.4%. Delta-positive variants had statistically lower MDI and PDI scores, according to the data. To better understand the long-term neurodevelopmental outcomes of SARS-CoV-2 (+) toddlers, we focused on this topic.

In the literature, there are limited and short-term studies assessing the neurodevelopmental outcomes of neonates infected with SARS-CoV-2 [13–14]. Poor neurodevelopmental outcomes were linked to birth during the SARS-CoV-2 pandemic rather than maternal SARS-CoV-2 infection, according to a study that compared infants with and without in utero exposure to maternal SARS-CoV-2 infection at 6 months of age [12]. In an Italian study, neonatal and short-term multidisciplinary outcomes were found to be normal, with the exception of ophthalmologic findings, in 199 asymptomatic newborns exposed to SARS-CoV-2 during pregnancy and the first hours of life [13]. Additionally, Mulkey et al. reported that on the 112th postnatal day, the neurological evaluations of infants born to symptomatic SARS-CoV-2 (+) mothers during the prenatal period were not normal. Their motor skills and language development scores were lower than those of babies born to asymptomatic mothers during pregnancy [14]. A recently published study that assessed relatively long-term neurodevelopmental outcomes in term toddlers at 16–18 months who had been exposed to SARS-CoV-2 in utero found a greater risk of neurodevelopmental impairment in these toddlers [15]. However, in all of these studies looking at neonatal SARS-CoV-2 exposure and neurodevelopmental outcomes, in utero SARS-CoV-2 exposure was examined, and short-term neurodevelopmental consequences were addressed. Conflicting results have been reported regarding the long-term consequences of exposure to SARS-CoV-2 in neonates. One study assessed the neurodevelopmental outcomes of newborns at 18 months who had in utero or postnatal exposure to SARS-CoV-2 infection and found no discernible difference between the study and control groups [11]. The SINEPOST project, on the other hand, sought to assess the neurodevelopmental outcomes of toddlers exposed to SARS-CoV-2 during prenatal or postnatal periods at postnatal 21–24 months; however, the study is still ongoing, and the findings have not yet been made public [36]. Approximately 75% of the toddlers in our study displayed symptoms of SARS-CoV-2 infection, although most of them had a mild course of the infection. Toddlers’ NDI scores were comparable to those of the control group, but their PDI scores were lower and their rates of MDPs were higher. This slight difference between the groups persisted with the addition of a low MDI score among newborns who were positive for the Delta variant. A Delta variant-positive toddler needed physical treatment, and another needed special education. These toddlers showed no symptoms of encephalitis, meningitis, or other neurological conditions during the study period. Even in the absence of meningitis, SARS-CoV-2 infection can cause brain damage and neurodevelopmental abnormalities [37]. While all of this is a consequence of the impact of undiagnosed long-term coronavirus disease (COVID) associated with neonatal SARS-CoV-2 infection, these results are areas open to investigation and attributable only to neonatal SARS-CoV-2 exposure. It is still unclear whether neonatal or maternal SARS-CoV-2 causes potentially dangerous long-term effects. Therefore, toddlers exposed to SARS-CoV-2 during the prenatal and neonatal periods must be watched carefully [11, 36, 37].

As one of our study’s secondary findings, we discovered that the infants’ head circumferences were smaller at the 12th month compared to the controls. Although it will eventually fade, this difference might help explain the fairly poor neurodevelopmental score. Longer hospital stays and shorter intervals of uninterrupted breastfeeding may also have had an effect on the mother-infant attachment process, leading to or exacerbating the effect of this minor neurodevelopmental score, regardless of whether these infants have had a SARS-CoV-2 infection. The importance of the mother-infant bonding process for the infant’s neurodevelopment has been shown in a number of studies to date. As a result, it is difficult to assign these findings primarily to neonatal SARS-CoV-2 infection [38, 39]. For informational purposes, this is the first study to evaluate the neurodevelopmental outcomes of 18–24-month-old children with SARS–CoV-2 infection and delta variant analysis in the early neonatal period.

Despite these strengths, our study contains a few drawbacks. The first drawback was that our study’s findings were limited to a single-center setting. The second restriction was the requirement for follow-up until school age to assess the academic performance of neonates infected with SARS-CoV-2. Third, there is untapped research potential in the area of vaccination defense and other safeguards against neurodevelopmental delays in neonates infected with SARS-CoV-2.

## Conclusions

Finally, our findings support the utility of neurodevelopmental screening in the long-term monitoring of SARS-CoV-2-positive neonates. At 18–24 months, SARS-CoV-2-positive neonates exhibited higher MDPs and lower PDI scores than unaffected neonates. In particular, the delta variant showed significantly lower MDI and PDI scores. Children should be reevaluated at ages 5 and 12 when they reach school age to discuss the true impact of SARS-CoV-2-positive neonates on long-term neurodevelopmental outcomes. To evaluate the true impact of SARS-CoV-2 infection in the neonatal era on long-term neurodevelopmental outcomes, prospective, multicenter studies with larger sample sizes are needed.

### Electronic supplementary material

Below is the link to the electronic supplementary material.

Supplementary Material 1

## Data Availability

The datasets generated and analyzed for the study are available from the corresponding author upon reasonable request.

## Abbreviations

Bayley: II Bayley Infant and Child Development Assessment Scale-II
CNS: Central Nervous System
COVID-19: Coronavirus disease 2019
MDI: Mental Development Index
MDP: Mildly Delayed Performance
MIS-C: Multi-System Inflammatory Syndrome in Children
NDI: Neurodevelopmental Retardation Index
NICU: Neonatal Intensive Care Unit
PDI: Psychomotor Development Index
SARS-CoV-2: Severe Acute Respiratory Syndrome Coronavirus-2
