# Supplementary material for: The long-term neurodevelopmental outcomes of toddlers with SARS-CoV-2 infection in the neonatal period: a prospective observational study
Source: Ital J Pediatr. 2024 Feb 27;50:34. doi: 10.1186/s13052-024-01609-w (PMC10900707; doi:10.1186/s13052-024-01609-w)
Supplement: Supplementary file 1 — Supplementary Material 1 [file 13052_2024_1609_MOESM1_ESM.docx]

**REVISION NOTE**

Dear Editor.

I would like to thank you very much for your consideration on our manuscript titled as “The Long-Term Neurodevelopmental Outcomes of Toddlers with SARS-CoV-2 Infection in the Neonatal Period: A Prospective Observational Study”. I also want to thank to the reviewers for their productive comments. In the light of their comments, I did the appropriate changes that are explained item by item in the subsequent page. You can find the highlighted changes within the text.

Thank you very much for your help.

Sincerely yours.

**ANSWERS FOR THE REVIEWERS’ COMMENTS.**

Changes within the manuscript are marked in red.

**Reviewer reports:**

**Reviewer #3:** I congratulate you on your work and especially on the topic you chose. Sars-Cov2 infection has had a significant clinical impact on our recent professional life; research is countless but unfortunately incomplete.

**I recommend shortening the abstract in order to make it quicker to read and to better emphasize the purpose of the study and its consequent clinical implications.**

**Answer:**

The abstract was shortened according to your suggestions.

**~~Abstract~~**

**~~Background~~** ~~The effect of Severe Acute Respiratory Syndrome Coronavirus-2 (SARS-CoV-2) infection in the neonatal period on the growing and developing brain is still unknown. This study aims to investigate the long-term neurodevelopmental outcomes of newborns exposed to SARS-CoV-2 & Delta variants.~~

**~~Methods~~** ~~At a tertiary referral center, a prospective observational cohort research was carried out. All babies who were equal to or more than 34 gestational weeks gestation and were admitted to the NICU between January 2021 and January 2022 due to SARS-CoV-2 infection (Delta - or Delta +) were included in the study. Infants who were hospitalized for non-SARS-CoV-2 reasons at similar dates and who had no history of invasive mechanical ventilation were incorporated as a control group using a 2:1 gender and gestational age match. Thirty infants were assigned to the study group and sixty newborns to the control group based on the sample size calculation. These toddlers' neurodevelopment was evaluated between the ages of 18 and 24 months using the Bayley-II scale.~~

**~~Results~~** ~~We enrolled 90 toddlers, including 30 toddlers in the study group (SARS-CoV-2 positive) and 60 toddlers in the control group (SARS-CoV-2 negative). Bayley-II scale results showed that SARS-CoV-2-positive toddlers had poorer psychomotor development index (PDI) scores and significantly greater mildly delayed performances (MDPs) at postnatal 18–24 months (PDI~~ *~~p=0.05~~*~~, MDPs~~ *~~p=0.03~~*~~, respectively). Low MDI or PDI scores were detected in 50% (n=15) of the SARS-CoV-2-positive toddlers. In the subgroup analysis, the delta variant showed significantly lower MDI and PDI scores (MDI~~ *~~p=0.03~~*~~, PDI~~ *~~p=0.03~~*~~, respectively). A smaller head circumference of SARS-CoV-2-positive toddlers was detected in the first year (~~*~~p<0.001~~*~~), which improved at the second age.~~

**~~Conclusion~~** ~~Neurodevelopmental findings of SARS-CoV-2-positive testing neonates revealed lower PDI scores and greater MDPs at the 18th-24th months when compared to unaffected controls. The effect is most noticeable in the delta variant. To explore the true impact on long-term neurodevelopmental outcomes and head circumference of SARS-CoV-2-positive neonates, it is critical to reevaluate these children between the ages of 5 and 12.~~

**~~Keywords:~~** ~~Neurodevelopment, Newborn, Bayley-II, SARS-CoV-2~~

**Abstract**

**Background** The effect of Severe Acute Respiratory Syndrome Coronavirus-2 (SARS-CoV-2) virus in the neonatal period on developing brain is still unknown. This study aims to investigate the long-term neurodevelopmental outcomes of newborns exposed to SARS-CoV-2 & Delta variant.

**Methods** At a tertiary referral center, a prospective observational cohort research was carried out. All babies who were equal to or more than 34 gestational weeks gestation and were admitted to the NICU between January 2021 and January 2022 due to SARS-CoV-2 infection (Delta - or Delta +) were included in the study. Infants who were hospitalized for non-SARS-CoV-2 reasons at similar dates and who had no history of invasive mechanical ventilation were incorporated as a control group using a 2:1 gender and gestational age match. Thirty infants were assigned to the study group and sixty newborns to the control group based on the sample size calculation. These toddlers' neurodevelopment was evaluated between the ages of 18 and 24 months using the Bayley-II scale.

**Results** We enrolled 90 infants. SARS-CoV-2-positive infants had poorer psychomotor development index (PDI) scores and significantly greater mildly delayed performances (MDPs) at 18–24 months (PDI *p=0.05*, MDPs *p=0.03*, respectively). Delta variant showed statistically significant lower MDI and PDI scores (MDI *p=0.03*, PDI *p=0.03*, respectively). A smaller head circumference of SARS-CoV-2-positive toddlers was detected in the first year (*p<0.001*), which improved at the second age.

**Conclusion** SARS-CoV-2-positive neonates revealed lower PDI scores and greater MDPs at 18th-24th months. The effect is most noticeable in Delta variant. Longer-term examination of neurodevelopmental outcomes and reevaluation of these children between the ages of 5 and 12 are critical.

**Keywords:** Neurodevelopment, Newborn, Bayley-II, SARS-CoV-2

**In addition, I think it may be useful to enrich the background with work on neonatal Sars-Cov2 infection. I point out this article, Falsaperla R, Giacchi V, Lombardo G, Mauceri L, Lena G, Saporito MAN, Vitaliti G, Palumbo MA, Ruggieri M. Neonates Born to COVID-19 Mother and Risk in Management within 4 Weeks of Life: A Single-Center Experience, Systematic Review, and Meta-Analysis. Am J Perinatol. 2021 Aug;38(10):1010-1022. doi: 10.1055/s-0041-1729557. Epub 2021 Jun 3. PMID: 34082444. I believe is useful in supplementing data on growth and outcome at one month of age in these children.**

**Answer:**

The systematic review and meta-analysis you suggested for the background part of the manuscript has been added.

Researchers are still monitoring what kind of symptoms SARS-CoV-2 infection produces in follow-up [10]. Currently, there is a lack of studies examining the impact of neonatal SARS-CoV-2 infection on the long-term neurodevelopment outcomes of affected infants. There are few studies on newborn neurodevelopment, particularly long-term studies [11]. Most of the follow-up studies are on infants born to mothers with SARS-CoV-2 infections [12-15]. In a study evaluating the routine management of newborns born to mothers with SARS-CoV-2 infection and their follow-up up to the first month, it was found that the risk of infection did not increase if the positive mother provided care to the newborn. It has been shown that breastfeeding and skin-to-skin contact, while adhering to protective measures, help babies' growth and development in the first month and even provide better results [new reference 16]. Some researchers describe that neonates born to mothers with SARS-CoV-2 infection or who had a SARS-CoV-2 infection in the early neonatal period have a favorable neurodevelopmental outcome [17 ~~16~~], while others reported neurodevelopmental sequelae and communication delay in some offspring [18 ~~17~~].

**New reference 16:** Falsaperla R, Giacchi V, Lombardo G, Mauceri L, Lena G, Saporito MAN et al. Neonates Born to COVID-19 Mother and Risk in Management within 4 Weeks of Life: A Single-Center Experience, Systematic Review, and Meta-Analysis. Am J Perinatol. 2021 Aug; 38 (10): 1010-1022. https://doi.org/10.1055/s-0041-1729557

**Reviewer #4:** First of all, congratulations.

The work is well written.

**The background is clear but the discussion on the neurological symptoms as well as the psychological effects on children of the Sars Cov-2 infection, already reported in the literature, could be expanded for example by inserting the citation of the article "Guido CA, Lucidi F, Midulla F, Zicari AM, Bove E, Avenoso F, Amedeo I, Mancino E, Nenna R, De Castro G, Capponi M, Cinicola BL, Brindisi G, Grisoni F, Murciano M, Spalice A; Long-Covid Group of Department of Maternal Sciences. Neurological and psychological effects of long COVID in a young population: A cross-sectional study. Front Neurol. 2022 Aug 17;13:925144. doi: 10.3389/fneur.2022.925144. PMID: 36062008; PMCID: PMC9428748." on line 68.**

**Answer:**

The literature you suggested for the discussion part of the manuscript (on line 68) has been added.

The SARS-CoV-2 virus has neuroinvasive properties. Adult investigations have indicated that some adults with moderate or severe SARS-CoV-2 infection have a predominance of neurological symptoms, increased neuronal damage, and glial activation markers [24-26 ~~23-25~~]. Evidence associating SARS-CoV-2 infection with brain abnormalities and cognitive problems in adulthood has since corroborated this theory [27-29 ~~26–28~~]. Studies have shown that long-term COVID findings (persistent fatigue, headaches, dyspnea, concentration issues, depression, skin lesions, and gastrointestinal complaints that persist months after initial SARS-CoV-2 infection) are also detected in pediatric populations, similar to the adult population, although the pediatric population has a milder course of SARS-CoV-2 infection than adults [30 ~~29~~]. In a survey study that involved 57 hospitals and 89 children with suspected COVID findings, fatigue was found in 45% of patients 2–12 months after acute infection, and severe functional limitations prevented children from attending school in 36% of patients [31 ~~30~~]. Anosmia, lethargy, and headaches were long-term, persistent COVID neurological symptoms in a research assessing the long-term clinical state of 322 SARS-CoV-2 positive children between the ages of 1.5 and 17 years. Furthermore, it was shown that 12% of the patients in the 1.5–5 age group experienced internalization issues, while the 6–18 age group experienced long-term psychological and cognitive issues such anxiety and post-traumatic stress disorder [new reference 32]. In a systematic review of 176 neonatal SARS-CoV-2 cases, researchers reported that 18% of infants had neurological signs such as hypertonia, irritability, as well as hypotonia and lethargy, and apnea [33 ~~31~~]. Additionally, in another report involving 5 neonatal SARS-CoV-2 cases, MRI showed various abnormalities in four; these were partially associated with reduced neurobehavioral scores [34 ~~32~~]. In order to evaluate the general movement assessment in infants 3-5 months old, Aldrete Cortez et al. videorecorded them. They compared 28 unexposed controls to 28 babies delivered to moms who infected SARS-CoV-2 during the third trimester. Babies in the exposed group saw a 22% incidence of absentee or less fidgety movements, which may be a sign of brain impairment early on [35 ~~33~~]. Our findings demonstrated that 50% of SARS-CoV-2-positive neonates had lower MDI or PDI scores. SARS-CoV-2-positive neonates additionally exhibited a 43% greater incidence of mild delay performance at the 18th-24th months than controls. Lower MDI or PDI scores were found in 81.8% of Delta-positive variants, while both lower MDI and PDI scores were found in 45.4%. Delta-positive variants had statistically lower MDI and PDI scores, according to the data. To better understand the long-term neurodevelopmental outcomes of SARS-CoV-2 (+) toddlers, we focused on this topic.

**New reference 32:** Guido CA, Lucidi F, Midulla F, Zicari AM, Bove E, Avenoso F et al. Neurological and psychological effects of long COVID in a young pupolation: A cross-sectional study. Front Neurol 2022 Aug 17; 13: 925144. https://doi.org/10.3389/fneur.2022.925144

The methods are explained correctly. The tables help understanding the text.
